# Effects of extraocular muscle surgery in children with monocular blindness and bilateral nystagmus

**DOI:** 10.1186/1471-2415-14-137

**Published:** 2014-11-20

**Authors:** Veit Sturm, Marketa Hejcmanova, Klara Landau

**Affiliations:** Department of Ophthalmology, University Hospital and University of Zurich, Zurich, Switzerland; Department of Ophthalmology, Cantonal Hospital St. Gallen, St. Gallen, Switzerland

**Keywords:** Monocular blindness, Manifest latent nystagmus

## Abstract

**Background:**

Monocular infantile blindness may be associated with bilateral horizontal nystagmus, a subtype of fusion maldevelopment nystagmus syndrome (FMNS). Patients often adopt a significant anomalous head posture (AHP) towards the fixing eye in order to dampen the nystagmus. This clinical entity has also been reported as unilateral Ciancia syndrome. The aim of the study was to ascertain the clinical features and surgical outcome of patients with FMNS with infantile unilateral visual loss.

**Methods:**

In this retrospective case series, nine consecutive patients with FMNS with infantile unilateral visual loss underwent strabismus surgery to correct an AHP and/or improve ocular alignment. Outcome measures included amount of AHP and deviation at last follow-up.

**Results:**

Eye muscle surgery according to the principles of Kestenbaum resulted in a marked reduction or elimination of the AHP. On average, a reduction of AHP of 1.3°/mm was achieved by predominantly performing combined horizontal recess-resect surgery in the intact eye. In cases of existing esotropia (ET) this procedure also markedly reduced the angle of deviation. A dosage calculation of 3 prism diopters/mm was established.

**Conclusions:**

We advocate a tailored surgical approach in FMNS with infantile unilateral visual loss. In typical patients who adopt a significant AHP accompanied by a large ET, we suggest an initial combined recess-resect surgery in the intact eye. This procedure regularly led to a marked reduction of the head turn and ET. In patients without significant strabismus, a full Kestenbaum procedure was successful, while ET in a patient with a minor AHP was corrected by performing a bimedial recession.

## Background

Three types of nystagmus in childhood can be distinguished according to the Classification of Eye Movement Abnormalities and Strabismus (CEMAS) Working Group. This group proposed the terms infantile nystagmus syndrome (INS) (old “congenital nystagmus”), fusion maldevelopment nystagmus syndrome (FMNS) (old “latent/manifest nystagmus”), and spasmus nutans syndrome [1].

Monocular infantile blindness or severe visual impairment may be associated with bilateral horizontal nystagmus. According to CEMAS classification, this form of nystagmus has been categorized under FMNS. It has been hypothesized that severe monocular visual impairment may act as an occluder, disclosing the otherwise latent nystagmus in patients who have a genetic predisposition for infantile esotropia (ET) [2]. Patients with FMNS with infantile unilateral visual loss form a subgroup within this entity, while FMNS is a common finding in children with infantile ET syndrome. In general, FMNS refers to a bilateral, conjugate, horizontal, uniplanar jerk nystagmus that occurs when one eye is occluded. The slow phase beats toward the covered eye. Usually there is no association with afferent visual abnormalities. Because FMNS and INS have some similarities and may exist simultaneously, they might be difficult to distinguish [1]. Differentiation can be made by eye-movement recordings. In FMNS two types of slow phases (linear and decelerating) are seen. In contrast, ocular motor recordings in INS typically show accelerating slow phases and a foveation period. The occurrence of an “adduction” dampening in FMNS, in which the eyes are in the position of least ocular instability and maximal visual acuity, is similar to the common null zone scenario in INS. Although this preferred eye position of minimal nystagmus intensity is quite typical, the underlying cause remains unclear. This specific fixation behaviour, which typically involves a nasalward drift of the fixing eye, is regularly accompanied by an anomalous head posture (AHP). Hence, patients often adopt a significant head turn towards the fixing eye in order to dampen the nystagmus, which typically increases on abduction and decreases on adduction. This clinical entity has also been reported in the literature as unilateral Ciancia syndrome [3–6]. It should also be differentiated from nystagmus blockage syndrome, which is often considered a special form of INS. In nystagmus blockage syndrome, a purposive ET either damps the INS or converts it to a low amplitude FMNS, thus improving visual acuity. The nystagmus reduction with the fixing eye in adduction and the head turn towards the fixating eye are similar to those in FMNS. However, the underlying pathophysiology is different. In FMNS, the ET and nystagmus are coincidental, whereas in nystagmus blockage syndrome the null zone in adduction precedes and causes ET. Accordingly, the ET increases as the nystagmus dampens, and decreases with increasing nystagmus [7].

Apparently, FMNS with infantile unilateral visual loss accounts for a considerable proportion of patients who assume AHP in order to dampen nystagmus. In Kushner’s series of 38 patients with AHP due to nystagmus, six patients had simultaneous congenital nystagmus and monocular blindness [8]. In another study on the oculographic and clinical characteristics of AHP in children, 32% of the included 37 children were diagnosed with a causative FMNS [9].

Surgery may be performed to improve visual acuity and/or to significantly reduce AHP. It has been suggested to direct extraocular muscle surgery to the fixing eye in order to prevent it from drifting in a nasalward direction. Besides this general idea, little is known how to approach these patients [10]. Accordingly, we set out to ascertain the clinical features and surgical outcome of patients with FMNS and pronounced AHP due to monocular infantile visual impairment or blindness.

## Methods

This was a retrospective case series of all consecutive patients with FMNS with infantile unilateral visual loss who underwent extraocular muscle surgery to eliminate an AHP and/or to improve ocular alignment. All patients were treated between January 1992 and December 2012 at the Strabismus and Pediatric Ophthalmology Service of the University Hospital of Zurich, Switzerland. This study adhered to the tenets of the Declaration of Helsinki. The Ethics Committee of the Canton of Zurich, Switzerland determined that ethics approval was not required since we performed a retrospective observational study of patients under our care.

### Parental consent section

Written parental informed consent was obtained from the patients’ parents for publication of case reports. A copy of the written parental consent is available for review by the editor of this journal.

### Ophthalmic and orthoptic examination

All patients underwent a complete assessment including medical history, ophthalmic and orthoptic examination. The ophthalmic examination included best corrected visual acuity measurement, slit-lamp assessment of the anterior segment, and dilated indirect binocular ophthalmoscopy. Cycloplegic refraction was performed after administration of 0.5% or 1% cyclopentolate (depending on the patients’/children’s ages) eye drops. Orthoptic examination included evaluation of ocular movements and angle of strabismus. Angles of deviation were measured using the Krimsky and the Hirschberg methods for near (1/3 m) and distance fixation. The Krimsky test was performed by placing a prism in front of the sound eye (modified Krimsky test).

The nystagmus pattern was clinically evaluated in all fields of gaze. For each eye, the directions of nystagmus were observed, i.e. the fast phase, amplitude and frequency (the product of which is the intensity) were evaluated. Any differences in the direction or intensity of movements between the eyes were used to distinguish between conjugate and disconjugate (dissociated) nystagmus.

Presence of pre- and post-operative AHP was ascertained at the Harms tangent screen while the patients were fixing the smallest possible target which was superimposed over the fixing light or using a simple surgical goniometer. Accordingly, the AHP was recorded in the vertical, sagittal and horizontal axes.

### Outcome measures

Patient demographics including gender, age at strabismus surgery, and length of follow-up, were analysed. Outcome measures included amount of AHP and deviation at last follow-up.

### Data analysis

Data were collected and analysed using the data analysis module from Microsoft Excel 7.0 (Redmond, WA).

## Results

Over the 19-year period, nine consecutive children (4 females, 5 males) with FMNS with infantile unilateral visual loss were identified. A jerk nystagmus was evident in all patients, consistent with a null zone towards the side of the pathologic eye. Four patients (patients 1, 4, 6, 9) demonstrated a rather simple AHP consisting of a head turn only, and the remaining five patients instead exhibited a more complex AHP. Three patients had adopted a combined head turn and head tilt (patient 2, 3, 5), while patient 8 displayed a combined head turn and chin down posture, and patient 7 had an AHP in all three planes. The mean head turn was 23° (range, 10-35°), while the mean head tilt was 15° (range, 0-20°). Except for the patient with the most complex AHP (patient 7), the head turn was generally the predominantly disturbing factor.

In the majority of cases (patients 4-9), the AHP was associated with a large ET. The mean angle of ET was 48 prism diopters (PD) (range, 35-55 PD) in these patients. The near and distance measurements were equal in all cases. In patient 7, ET was accompanied by a left hypertropia of 25 PD. The remaining three cases had a cosmetically acceptable alignment, i.e. patient 1 had 8 PD near exotropia, patient 2 was orthotropic and patient 3 showed a small exotropia of 10 PD.

The mean age at surgery was 8.7 years (range, 2-18 years).

The clinical data for each patient are summarized in Table 1.

**Table 1:** **Ocular findings in patients with FMNS with infantile unilateral visual loss**

Patient/Sex/Age at surgery ***(years)***	Cause of monocularity	Pre-operative situation				Surgical Procedure ***(millimeters)***	Post-operative situation				Follow up***(months)***
		BCVA ***(with optimum head posture)***	Characteristics of Nystagmus	Abnormal head posture ***(Degrees)***	Eye alignment ***(D/N, prism diopters)***		BCVA ***(with optimum head posture)***	Characteristics of Nystagmus	Abnormal head posture ***(Degrees)***	Eye alignment ***(D/N, prism diopters)***	
**1**/M/10	L: congenital corneal opacity	R: 20/20	jerk nystagmus, null zone in left gaze	18° right face turn	0/-8	R: MR recess 5/LR resect 8	R: 20/20	jerk nystagmus, null zone in PP	none	0/0	3
		L: 20/4000				L: MR resect 6/LR recess 7	L: 20/4000				
**2**/M/8	L: corneal dermoid	R: 20/25	jerk nystagmus, null zone in left gaze	15° right face turn, 10° right head tilt	0/0	R: MR recess 7/LR resect 10	R: 20/25	jerk nystagmus, null zone in slight left gaze	5° right face turn, 8° right head tilt	0/0	12
		L: NLP					L: NLP				
**3**/M/6	L: congenital	R: 20/30	jerk nystagmus, null zone in left gaze	35° right face turn,15° right head tilt	-10/-10	R: MR recess 6/LR resect 8	R: 20/25	jerk nystagmus,	none	0/0	23
	microphthalmus	L: LP				L: MR resect 6/LR recess 8	L: LP	null zone in PP			
**4**/F/2^1^	R: optic nerve hypoplasia	R: 20/200	jerk nystagmus, null zone in right gaze	20° left face turn	+35/+35	R/L: MR recess 4.5	R: 20/200	jerk nystagmus, null zone in PP	none â€“ 10° left face turn	0/0	
		L: 20/20					L: 20/20				
	R: optic nerve hypoplasia		jerk nystagmus, null zone in right gaze	25° left face turn	+4/+4	R: LR recess 5.5 L: MR recess 5.5		jerk nystagmus, null zone in slight right gaze	12° left face turn	-5/-5	202
**5**/F/5^2^	R: optic nerve hypoplasia	R: LP	jerk nystagmus, null zone in right gaze	35° left face turn, 15° left head tilt	+55/+55	L: MR recess 6/LR resect 8	R: LP	jerk nystagmus, null zone in PP	15° left head tilt	+20/+25	
		L: 20/20					L: 20/20				
	R: optic nerve hypoplasia	R: LP	jerk nystagmus, null zone in right gaze	20° left face turn, 15° left head tilt	+35/+35	R: MR recess 6/LR resect 6	R: LP	jerk nystagmus, null zone in PP	10° left head tilt	0/+6	60
		L: 20/25					L: 20/20				
**6**/F/18	L: congenital cataract and microphthalmus	R: 20/20	jerk nystagmus, null zone in left gaze	12° right face turn	+45/+45	R: MR recess 5/LR resect 7	R: 20/20	jerk nystagmus, null zone in PP	none	0/0	3
		L: LP					L: LP				
**7**/M/16	R: congenital microphthalmus	R: NLP	jerk nystagmus, null zone in right and up-gaze	10° left face turn 20° right head tilt 15° chin down posture	+45/+45	L: SO tuck 9 mm	R: NLP	null zone in PP	5° right head tilt	0/0	12
		L: 20/20			L/R 25	IO anteroposition	L: 20/20				
						LR resect 8 and full tendon width downward shift					
**8**/F/9	R: congenital microphthalmus	R: LP	jerk nystagmus, null zone in right gaze	30° left face turn, 3° chin down posture	+55/+55	L: MR recess 6.5 / LR resect 10	R: LP	jerk nystagmus, null zone in PP	5° left face turn	+8/+8	3
		L: 20/25					L: 20/25				
**9**/M/4	L: retinal hamartoma	R: 20/25	jerk nystagmus, null zone in left gaze	35° right face turn	+55/+55	R: MR recess 6/LR resect 8	R: 20/25	jerk nystagmus, null zone in left gaze	20° right face turn	+8/+20	12
		L: 20/400					L: 20/400				

D: Distance, N: Near, M: male, L: left, R: right, MR: medial rectus, LR: lateral rectus, PP: primary position, F: female; +: esotropia, -: exotropia.

^1^reoperation 5 years later.

^2^reoperation 13 months later.

Two patients are described in detail below to better illustrate the different preoperative situations and their management.

### Patient 5

A 5-year-old girl presented with a right ET of about 55 PD at both distance and near due to severe optic nerve hypoplasia in the right eye, while the left eye appeared structurally normal. Ophthalmic examination disclosed a manifest nystagmus of the latent type, i.e. a rapid jerk nystagmus beating leftward in both eyes. The nystagmus was reduced by left fixation in extreme adduction. The girl had adopted a combined AHP with a left head turn (35°) and left tilt (15°), which allowed her to use her null zone in right gaze. The patient underwent combined horizontal surgery in her better-seeing left eye (6-mm recession of the medial rectus muscle [MR] and 8-mm resection of the lateral rectus muscle [LR]). Postoperatively, her ET was reduced to 20 PD at distance and 25 PD at near. The AHP was significantly improved, decreasing to a left head tilt of 15°.

One year later, a second combined horizontal surgery was performed on the structurally abnormal right eye to treat cosmetically unacceptable ET (MR recession of 6 mm and LR resection of 6 mm). At the last follow up one year later, the patient tilted her head only slightly to the left (10°). She had maintained a satisfactory ocular alignment (orthotropic at distance, 6 PD ET at near).

### Patient 7

A 16-year-old boy with a prosthetic right eye due to congenital microphthalmus (with a visual acuity of no light perception) was referred to our department. Visual acuity was 20/20 in the normal left eye. He had adopted a complex AHP posture to minimize the left-beating jerk nystagmus. The AHP included a 10° left head turn, a 20° right head tilt and a 15° chin down posture. The Krimsky test (performed in the patient not wearing the prosthesis) revealed a 45 PD ET and a left hypertopia of 25 PD. Extraocular muscle surgery (left MR recession of 10 mm) had been performed elsewhere 14 years ago at the age of 2 years because of strabismus (30 PD ET and 35 PD left hypertropia) and a similar AHP. The initial AHP, as measured before the first surgery, was recorded as 20° left head turn, mild right head tilt, and chin down posture.

Due to the complex situation, we performed a second strabismus surgery in the left eye to address the AHP and the misalignment (superior oblique tendon tuck of 9 mm, inferior oblique anteroposition of 10 mm, 8-mm resection of the LR and full tendon width downward shift of muscle insertion). One year after surgery the AHP was markedly reduced with 5° right head tilt remaining.

### Surgical procedures

In five cases a combined horizontal recess-resect surgery of the intact eye was initially performed (patients 2, 5, 6, 8, 9). On average the MR was 6.1 mm recessed (range, 5-7 mm) and the LR 8.6 mm resected (range, 7-10 mm). Two patients (patients 1, 3) without significant strabismus had a recess-resect surgery of all four horizontal rectus muscles. The amount of surgery was approximately similar (26 mm and 28 mm, respectively). In patient 4, a bilateral recession of the MR was initially performed. Patient 7, who had an initial MR recession in the intact eye elsewhere, underwent a more complex surgery in our hands (superior oblique tendon tuck, inferior oblique anteroposition, resection of the LR and full tendon width downward shift of muscle insertion) as described above.

Reoperations were performed in two of the cases due to recurrence of the head turn (patients 4, 5) and residual ET (patient 5). In patient 4 an Anderson procedure (right LR recession and left MR recession) was performed as second surgery, while patient 5 received another recess-resect surgery in the eye with the optic nerve hypoplasia.

No surgical complications were observed, i.e. neither motility deficits nor lid malpositions occurred.

### Postoperative outcome

A combined recess-resect surgery of the intact eye was performed as the standard procedure. A marked reduction of the AHP was obtained in all five cases (mean: 19°, range, 10-35°).

Accordingly, a mean reduction of head turn of 1.3°/mm was calculated. When present (patients 5, 6, 8, 9), the associated ET was also significantly reduced (mean: 44 PD, range, 35-47 PD for ET at distance).

Overall, the combined recess-resect surgery had a mean effect of 3 PD/mm on ocular alignment. The residual head turn ranged from 0° to 20° (mean: 6°) and the residual mean ET was 9 PD (range, 0-20 PD). In patient 5, a reoccurrence of the head turn (20°) and a slight increase of the residual ET was observed. The re-operation (recess-resect surgery of the fellow eye) abolished the head turn and ET.

In patients 1 and 3 a full Kestenbaum procedure was performed. In both cases surgery was performed to correct the head turn as misalignment was negligible. The head turn was eliminated in both patients (18° reduction and 35° reduction, respectively).

In patient 4 bilateral MR recession (4.5 mm) was performed as the ET (35 PD) seemed to be the more disturbing factor. The ET was completely corrected and the head turn was reduced from 20° to 10° as well. Over time, an increase of the AHP was observed. Accordingly, a reoperation (right LR recession 5.5 mm and left MR recession 5.5 mm) was performed five years later, which reduced the head turn from 25° to 12°.

The mean follow-up after surgery was 39 months (range, 3-202 months).

## Discussion

Surgical therapy in nystagmus must be tailored according to the underlying type and the specific features associated. Extraocular muscle surgery for INS is indicated to correct head turn by moving an eccentric null point closer to the primary position or to treat associated strabismus. Surgery can also improve INS waveform characteristics [11]. Surgery for nystagmus blockage syndrome is aimed at reducing the head turn. Bilateral MR posterior fixation sutures, bimedial recession (with or without posterior fixation sutures), or unilateral recess-resect surgery have been suggested [12]. Spasmus nutans syndrome typically resolves spontaneously and does not require specific treatment. Primary treatment of FMNS is rather conservative and consists of measures to improve visual acuity and binocular function. Surgical therapy has to take into account the different aspects of FMNS. Surgery may be performed to correct the AHP and strabismus and might enhance visual acuity as well. In our patients with FMNS with infantile unilateral visual loss, surgery was predominantly indicated due to the associated pronounced AHP.

A general approach in such cases of AHP, regardless of the underlying cause, is to place the eyes into the null zone. Anderson, Goto and Kestenbaum are generally considered the pioneers in the field of surgical management of nystagmus. In the early 1950s, they independently suggested to shift the eyes in the direction of the head turn, and away from the preferred direction of gaze [13–15]. The rationale is to create a gaze palsy to the side of preferred fixation. Anderson proposed a recession of the horizontal yoke muscles acting as agonists in the direction of the head turn, while Goto described a resection of the antagonistic muscle of each eye. Kestenbaum instead advocated surgery on all four horizontal rectus muscles. In 1973, Parks published his popular “5-6-7-8” guideline. A total of 13 mm surgery was proposed for each eye: 5-mm recession of the MR and 8-mm resection of the LR for the adducted eye and 6-mm resection of the MR and 7-mm recession of the LR for the fellow eye [16]. Due to frequent undercorrections and recurrences of the head turn, modified and augmented surgery was later suggested [17–22].

While Kestenbaum surgery, with its alterations, has become a well-established treatment for patients with nystagmus and horizontal head turns, its role in vertical AHP and head tilts is not as well understood. The scarce number of studies were often limited by small numbers of patients and short follow-up periods [23–26]. The basic principle is the same as in horizontal AHP and follows the general idea of shifting the null point towards primary gaze position.

In the surgical management of our patients it was important to consider that they had only one intact eye. In such cases the widely accepted approach of shifting the null zone to the primary position, usually obtained by changing the position of both eyes in the orbit, has to be modified. Therefore, in patients with strabismus and/or poor vision in one eye it has been emphasized to direct the surgery to the fixing eye [10]. Since patients often are reluctant to undergo surgery on their better eye, it can be helpful to demonstrate the expected effects of surgery by means of injection of botulinum toxin to the MR of the dominant eye [27].

In the majority of our cases surgery was primarily performed to correct the bothersome head turn. We performed a combined recess-resect surgery in the better eye as the standard procedure. We used a moderate amount of eye muscle surgery, i.e. the MR was 6.1 mm recessed on average and the LR was 8.6 mm resected on average. This led to an average reduction of the head turn by 19° (mean reduction of head turn of 1.3°/mm). When present, the associated ET was also reduced. An effect of 3 PD/mm was calculated for the combined recess-resect surgery. This pronounced effect on alignment has not received much attention yet.

In two cases without significant strabismus, we were able to correct the head turn by applying the full Kestenbaum procedure. Even with a bilateral MR recession as performed in one patient to mainly correct the ET, a 10° reduction of the head turn occurred.

Patients with FMNS and monocular infantile blindness can also adopt complex AHP involving all three planes. In our case a major reduction of the AHP (10° left head turn, 20° right head tilt and 15° chin down posture) was achieved with a superior oblique tendon tuck of 9 mm, inferior oblique anteroposition of 10 mm and 8-mm resection of the LR including a full tendon width downward shift of the muscle insertion.

No surgical complications were observed, i.e. none of the patients developed motility deficits or lid malpositions. However, two cases required reoperation. In one patient the head turn had reoccurred and the other patient underwent a second surgery due to the recurrence of the head turn and residual ET.

To the best of our knowledge, only few studies on the surgical management of FMNS have been published [2, 10, 27, 28]. They predominantly offered general recommendations how to approach these patients [2, 10]. In patients with FMNS and uniocular blindness, a combined horizontal recess-resect surgery in the sound eye was advocated to correct the AHP [2, 28]. Lee instead favoured a graded approach [10]. In patients with a head turn without strabismus, a recession and posterior fixation of the MR of the dominant eye was suggested to prevent the eye from drifting in a nasalward direction. In patients with AHP and ET, a combined recess-resect surgery on the dominant eye was proposed.

However, only small case series discussing surgical results have been published [27, 28]. Previous reports lacked details on clinical picture and follow-up. Liu *et al*. discussed the surgical management of a female patient [27]. She received a recess-resect surgery on her only eye that improved the visual acuity in primary position; however, detailed information on AHP was not provided. Although Helveston performed a recess-resect surgery in a two-year-old monocular boy, which improved the AHP, this report lacked exact data [28]. In another monocular patient, a single MR recession was done with success, yet a more detailed discussion is missing.

Our results confirm the general surgical approach to correct AHP. In FMNS, patients adopt their AHP to fixate in adduction to decrease nystagmus amplitude. Since the AHP is driven by the sound (dominant) eye, surgery according to the principles of Kestenbaum, Anderson and Goto has to be performed on the fixating eye shifting the null zone towards the viewing eye.

However, we advocate a tailored surgical approach in patients with FMNS with infantile unilateral visual loss. In patients adopting a significant head turn accompanied by a large esotropia we suggest an initial combined recess-resect surgery in the intact eye, which regularly led to a marked reduction of the head turn and the ET. This pronounced effect on alignment has not received much attention yet. Our surgical numbers might be used as rough guidelines. The combined recess-resect surgery resulted in a mean effect of 3 PD/mm on ocular alignment and a mean reduction of head turn of 1.3°/mm. It seems that the response to surgery was somewhat correlated with the preoperative AHP. Hence, the self-adjusting effect of the procedure may contribute to the success.

In patients without significant coexisting strabismus a full Kestenbaum procedure might be performed. A recession and posterior fixation of the MR of the dominant eye, as suggested by Lee *et al*., could be done as well [10]. A bimedial recession might be applied in FMNS with marked ET and less AHP. In cases of existing or residual tropia, the surgery might also be performed on the structurally abnormal eye.

## Conclusions

We advocate a tailored surgical approach in FMNS with infantile unilateral visual loss. Extraocular muscle surgery might be performed to improve AHP and strabismus. In typical patients who adopt a significant head turn accompanied by a large ET, we suggest an initial combined recess-resect surgery in the intact eye. This procedure regularly led to a marked reduction of the head turn and the ET. A full Kestenbaum procedure was successful in patients without significant existing strabismus, while a bimedial recession corrected the ET in a patient with a small AHP.
